# Characteristics of Autism Spectrum Disorder in Sotos Syndrome

**DOI:** 10.1007/s10803-016-2941-z

**Published:** 2016-10-22

**Authors:** Chloe Lane, Elizabeth Milne, Megan Freeth

**Affiliations:** Department of Psychology, The University of Sheffield, Western Bank, Sheffield, S10 2TP UK

**Keywords:** Sotos syndrome, Autism spectrum disorder, Behaviour, Social responsiveness scale

## Abstract

Sotos syndrome is a congenital overgrowth disorder with an incidence of approximately 1 in 14,000. This study investigated behavioural characteristics of ASD within a large cohort of individuals with Sotos syndrome (n = 78). As measured by the Social Responsiveness Scale, second edition (SRS-2), 65 participants (83.33 %) met clinical cut-off (T-score ≥60). There was no significant gender difference in symptom severity. There was a significant effect of age, with lower scores observed in early childhood and adulthood, compared to childhood. Furthermore, individuals with Sotos syndrome appear to display a trait profile that is similar to that identified in ASD. Overall, these findings indicate that the majority of individuals with Sotos syndrome display clinically significant behavioural symptomatology associated with ASD.

## Introduction

Autism spectrum disorder (ASD) is a developmental disorder associated with social communication impairment and restricted interests and repetitive behaviours. The disorder occurs in approximately 1 % of the population (Baird et al. 2006; Baron-Cohen et al. 2009). ASD symptomatology has been reported in a number of congenital syndromes, including Fragile X (Kaufmann et al. 2004), Cornelia de Lange (Moss et al. 2012) and Angelman syndrome (Peters et al. 2004). It has been suggested that approximately 10–20 % of cases of ASD are caused by genetic syndromes, cytogenetics lesions and rare de novo mutations (Abrahams and Geschwind 2008). Consequently, a number of aetiological genetic pathways may be implicated in ASD (Abrahams and Geschwind 2008; Zhao et al. 2007). Thus, investigation of the association between ASD and genetic syndromes is particularly valuable in identifying genetic mechanisms associated with ASD. Furthermore, distinct ASD phenotypes may be associated with each genetic syndrome (Moss and Howlin 2009). It is therefore important to establish the profile of autistic symptomatology within a syndrome as this will facilitate understanding of both autism and genetic syndromes.

Sotos syndrome is a congenital overgrowth disorder which was initially recognised in 1964 (Sotos et al. 1964). The estimated incidence is 1 in 14,000 (Tatton-Brown and Rahman 2004). Diagnostic criteria include overgrowth with advanced bone age, macrocephaly, characteristic facial appearance and intellectual disability (Cole and Hughes 1994). Haploinsufficiency of the NSD1 (nuclear receptor binding SET domain protein 1) gene was first identified as the primary cause of Sotos syndrome in 2002 (Kurotaki et al. 2002). This gene encodes SET domain-containing histone methyltransferases and is located at chromosome 5q35 (Tatton-Brown and Rahman 2013). Sotos syndrome is caused by intragenic mutations or microdeletions of the NSD1 gene, resulting in loss of function and it has been suggested that abnormalities of the NSD1 gene are present in more than 90 % of individuals with a clinical diagnosis of Sotos syndrome (Tatton-Brown et al. 2005). The aim of the present study was to investigate the prevalence and profile of ASD characteristics within a large cohort of individuals with Sotos syndrome.

A recent systematic review and meta-analysis investigated the prevalence of reported ASD symptomatology in a range of genetic syndromes (Richards et al. 2015). Twelve syndromes were included in this review and a quality-weighted effect prevalence was generated for each of the syndromes. This was based on the reported prevalence of ASD in the relevant studies for each of the syndromes and adjusted, based on the quality ratings of the studies. A quality checklist was generated by the authors using existing standardised quality criteria for intervention and prevalence studies. Higher quality studies received greater weighting in the prevalence estimates. The prevalence estimates of the number of individuals who met clinical cut-off for ASD ranged from 11 % in 22q11.2 deletion syndrome to 61 % in Rett’s syndrome and all twelve syndromes had a prevalence estimate significantly above that of the general population. Thus, this review provides evidence for increased prevalence of ASD symptomatology in genetic syndromes and suggests significant variability in prevalence between syndromes. Sotos syndrome was not included in this review due to a lack of previous research investigating the prevalence of ASD in Sotos syndrome. However, as Sotos syndrome has a genetic cause, it is important to establish the prevalence of ASD within this population in order to determine whether the NSD1 gene could be implicated in ASD.

Comparison of the profiles of ASD symptomatology in distinct syndromes is beneficial in advancing understanding of the specific behavioural profile associated with a particular syndrome. This is particularly useful for identifying areas in which to target interventions. Van Eeghen et al. (2013) used a cross-disorder approach to investigate relationships between ASD and several biologically related disorders: tuberous sclerosis complex (TSC), neurofibromatosis type 1 (NF1) and childhood-onset epilepsy of unknown cause (EUC). Sotos syndrome was not included in this study as it is not associated with mutations in a tumour-suppressor gene and is therefore not biologically related to TSC, NF1 or EUC. Autistic features were assessed using The social responsiveness scale (SRS) (Constantino and Gruber 2005) which provides a quantitative measure of ASD symptomatology. The findings from this study suggest that each of the disorder groups displayed a trait profile similar to that of ASD, specifically in relation to difficulties in social cognition and repetitive mannerisms, but at a lower severity level. Although some disorders display similar trait profiles to that of ASD, some congenital syndromes are associated with subtly different profiles of ASD symptomatology (e.g. Cornelia de Lange Syndrome; Moss et al. 2012). It is therefore important to explore the trait profile of ASD symptomatology within Sotos syndrome in order to establish whether the behavioural profile is similar or distinct to that of idiopathic ASD.

In a systematic review of the published literature on cognition and behaviour in Sotos syndrome, we identified a potential link between Sotos syndrome and ASD (Lane et al. 2016). Our review identified four studies which have provided data relating to Sotos syndrome and ASD. Of these, three were case studies of individuals who had co-morbid diagnoses of Sotos syndrome and ASD. Mouridsen and Hansen (2002) reported a case of a young child with Sotos who met the ICD-10 diagnostic criteria for childhood autism. Morrow et al. (1990) reported a child with Sotos syndrome who, following clinical observation, was reported to meet diagnostic criteria for ASD. Additionally, Trad et al. (1991) reported a case of a child with Sotos syndrome who met DSM-III-R criteria for pervasive developmental disorder. In addition to these case studies, Zappella (1990) reported a case series of 12 children with Sotos syndrome. The aim of this study was to investigate the prevalence of autistic features in each of these 12 children, using behavioural observation. Within this sample, the authors noted that 5 children (41.67 %) displayed autistic features consistent with the DSM-III-R criteria for autistic disorders. While this study suggests that the incidence of ASD in Sotos syndrome is greater than in the general population, the small sample size means that it is not possible to establish the prevalence of ASD within the Sotos population as a whole.

Timonen-Soivio et al. (2016) recently investigated the relationship between ASD and Sotos syndrome in a cohort of Finnish children. Population registers were searched in order to identify the number of individuals with co-morbid diagnoses of distinct congenital syndromes and ASD. The study identified a significant association between ASD and Sotos syndrome. Of the 13 children identified with Sotos syndrome, 7 (53.85 %) had a co-morbid diagnosis of ASD. Therefore, this study provides further evidence for an increased prevalence of ASD within the Sotos population but again, the sample size is small. In addition, this study assessed the relationship between ASD and Sotos syndrome in terms of co-morbid diagnoses and therefore autistic symptomatology was not explicitly measured within this study. It is possible that further individuals with Sotos syndrome may display behaviour that would meet diagnostic criteria for ASD but had not received a formal diagnosis.

In a recent study, Sheth et al. (2015) reported characteristics of ASD in a sample of 38 individuals with Sotos syndrome, as assessed by the social communication questionnaire (SCQ) (Rutter et al. 2003) and the repetitive behaviour questionnaire (RBQ) (Moss and Oliver 2008). Mean age of the participants was 17.3 years, with an age range of 6–43 years. The SCQ is a standardised 40-item questionnaire, designed to assess symptomatology associated with ASD. There are three SCQ subscales (reciprocal social interaction; communication; restricted, repetitive and stereotyped patterns of behaviour) which are based on the DSM-IV criteria for ASD. There are two versions of the SCQ: a Current form and a Lifetime form. Sheth et al. (2015) used the Lifetime form which is concerned with both behaviours that have been present at any point in the individual’s life, as well as behaviours that occurred during a 12 month period (4–5 years of age). Consequently, the Lifetime form has a significant focus on the period of development during the ages of 4 and 5 years and is therefore not an appropriate measure to compare changes in symptomatology over time.

Sheth et al. (2015) found that 26 of 38 participants with Sotos syndrome (68.42 %) met clinical cut-off for ASD, as measured by total score on the Lifetime version of the SCQ (clinical cut-off was considered as a total score ≥15). Data from the Sotos syndrome group were compared with data from three distinct, matched control groups: ASD, Prader-Willi syndrome and Down syndrome. Participants with Sotos syndrome scored significantly lower than the ASD group on the repetitive behaviour subscale of the SCQ but there were no significant differences between the Sotos and ASD groups on the social communication and social interaction subscales. Subsequent analyses using only the Sotos participants who scored above clinical cut-off, identified no significant differences between the Sotos and ASD groups for the three SCQ subscales. The RBQ is a 19-item questionnaire, designed to assess behaviours across five subscales: restricted preferences, repetitive speech, insistence on sameness, stereotyped behaviour and compulsive behaviour. No standardised norms or clinical cut-off are available for this measure. However, when compared to an ASD group, the Sotos syndrome group scored significantly lower than the ASD group on the stereotyped behaviour subscale but there were no significant differences between scores on the remaining subscales between the Sotos and ASD participants. Overall, the findings from this study suggest that a high proportion of individuals with Sotos syndrome display autistic characteristics of a clinical nature. Difficulties associated with repetitive behaviour are less severe than observed in ASD for individuals with Sotos who do not score above clinical cut-off, despite significant impairment in social communication and social interaction. As this study used the Lifetime version of the SCQ, some of the questions focus on the developmental period of 4–5 years of age so it is therefore not currently known whether these reported difficulties also apply to later childhood and adulthood.

The current study complements and extends the findings from Sheth et al. (2015) in a number of important ways. Based on previous literature, the variability of ASD symptom severity within the Sotos population is not clear and a detailed profile analysis of ASD symptomatology has not been established. In addition, the effects of age and gender on symptom severity have not been explored. Here, we investigate the prevalence of symptoms associated with ASD in a larger sample (n = 78), using a measure of ASD symptomatology that is consistent with the DSM-5 criteria for ASD diagnosis—(Social Responsiveness Scale, second edition; SRS-2) (Constantino and Gruber 2012). The SRS-2 provides a quantitative measure of autistic symptomatology and is designed to measure severity of deficit in reciprocal social interaction, as well as deficit in restricted interests and repetitive behaviours. Scores are categorised as non-clinical, or as indicative of mild, moderate or severe issues with reciprocal social interaction. To date, this measure has not been used to investigate quantitative, intragroup autistic features in Sotos syndrome. An additional benefit of the SRS-2 is that, by providing T-scores, it is possible to compare data from males and females and from different age groups. Furthermore, a recent factor analysis (Frazier et al. 2014) identified five empirically derived factors that can be assessed using the SRS-2: emotion recognition, social avoidance, interpersonal relatedness, insistence on sameness and repetitive mannerisms. These additional factors can be used to explore the profile of ASD symptomatology. The SRS-2 can also be used to investigate effects of gender and age (Frazier et al. 2014) on ASD symptomatology and these factors have not yet been explored within the Sotos population.

The primary aims of this study were to identify the prevalence of autistic features within a large cohort of individuals with Sotos syndrome and to explore the profile of autistic features within this population. It was hypothesised that a significant proportion of individuals with Sotos syndrome would score above clinical cut-off for ASD symptomatology. Secondary aims of this study were to investigate differences in symptom severity in relation to gender and age.

## Method

### Participants

The SRS-2 was completed by a family member for 78 individuals with a diagnosis of Sotos syndrome (see Table 1 for participant characteristics). Families were recruited via the Child Growth Foundation (CGF; a UK charity that supports families of individuals affected by growth disorders) and advertisements on Sotos syndrome support groups on social media. Specifically, the research was advertised on two Facebook groups: ‘Sotos Syndrome—UK’ and ‘Sotos Syndrome/Cerebral Gigantism’ as a ‘personality and behaviour study’. ASD was not mentioned in the study information, in order to avoid biasing the sample. All respondents were asked to complete a screening form, in order to establish eligibility for the study. Families were asked to state whether their child or partner had been diagnosed with any developmental disorders and if so, to list these. Any families who did not list Sotos syndrome were excluded. One family was excluded as they reported that their child had ‘reverse Sotos syndrome’ and one family was excluded on the basis that their child had ‘suspected Sotos syndrome’ but a diagnosis of Sotos syndrome had not yet been confirmed by a clinician. As well as reporting a diagnosis of Sotos syndrome, some respondents reported that their child or partner also had a diagnosis of ASD (n = 16), an anxiety disorder (n = 10) or ADHD (n = 4).

**Table 1:** Participant characteristics

Characteristics	Participants (n = 78)
Age (in years)	
Mean	12.13
SD	8.99
Range	2.5–50
Gender (n)	
Males	43
Females	35
Nationality (n)	
British	40
American	18
Other	20

### Measures

The SRS-2 is a 65-item questionnaire with each item being coded on a Likert scale (0 = not true to 3 = almost always true), designed to assess symptoms associated with ASD. A total score indicates severity of ASD symptomatology, with a higher score indicating greater severity. The SRS-2 has a conceptually derived two-factor structure that is consistent with the DSM-5 criteria for ASD. The factors are social communication impairment and restricted interests and repetitive behaviours. The SRS-2 has been found to be a valid measure of autistic symptomatology across cultures (Bölte et al. 2008; Wigham et al. 2012). Previous research has identified that scores on the SRS-2 are not related to intelligence (Charman et al. 2007) or age (Bölte et al. 2008). A recent confirmatory factor analysis (Frazier et al. 2014) identified an additional five SRS-2 specific factors: emotion recognition, social avoidance, interpersonal relatedness, insistence on sameness and repetitive mannerisms.

Age appropriate versions of the SRS-2 were used; pre-school (2.5–4 years; n = 15), school age (4–18 years; n = 46) and adult (19 years and older; n = 17) and the questionnaire was completed by either the parent/caregiver (n = 76), other specialist (n = 1) or spouse (n = 1) of each participant. All questionnaires were completed in English. Licensing was received by the publishers of the SRS-2 to allow online administration of the questionnaire. The study received ethical approval from the university Departmental Ethics Committee.

## Results

### Clinical Cut-off

**Fig. 1:**
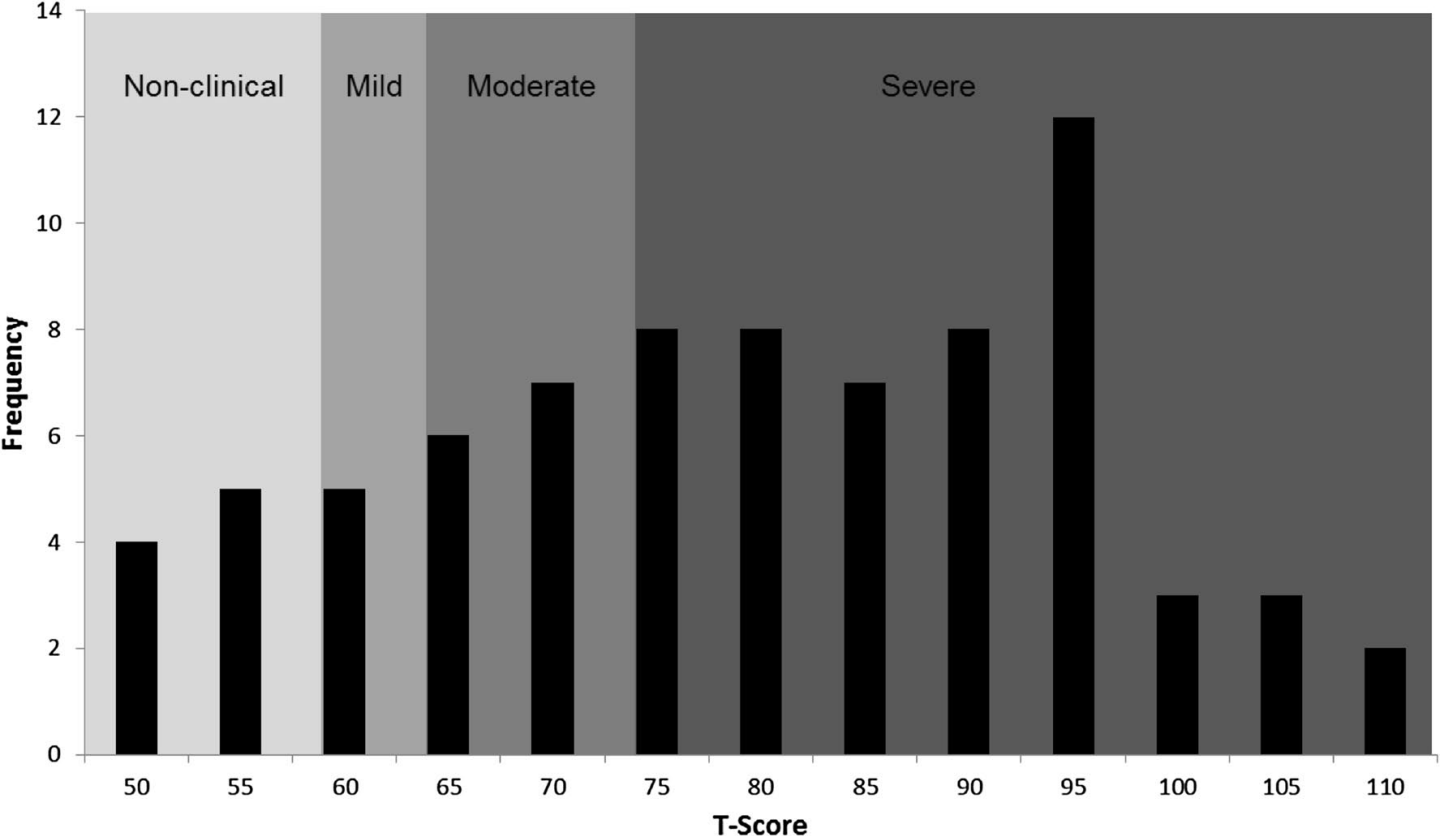
Distribution of total SRS-2 T-scores

Clinical cut-off was considered as a total T-score ≥60 (Constantino and Gruber 2012). The mean T-score of this group of 78 individuals was 77.13 (SD = 15.91) and 65 participants (83.33 %) met clinical cut-off for behavioural symptomatology associated with ASD (Fig. 1). All participants with diagnoses of both Sotos syndrome and ASD (n = 16) scored above clinical cut-off. Within the total sample, 55.13 % (n = 43) were in the severe clinical range (T-score ≥76), 19.23 % (n = 15) were in the moderate clinical range (T-score of 66–75) and 8.97 % (n = 7) of scores were in the mild clinical range (T-score of 60–65). Total T-scores ranged from 44 to 109. Data were normally distributed.

### Gender Differences

In order to establish whether gender affects ASD symptom severity within the Sotos population, an independent samples *t* test was used to compare total T-scores in male and female participants. The analysis identified no significant difference (*t*(77) = 0.93, *p* = .926) in total T-scores for male (M = 76.98, SD = 14.61) and female (M = 77.31, SD = 17.59) participants. This suggests that within the Sotos population, there are no gender differences in ASD symptom severity.

### Age Differences

**Fig. 2:**
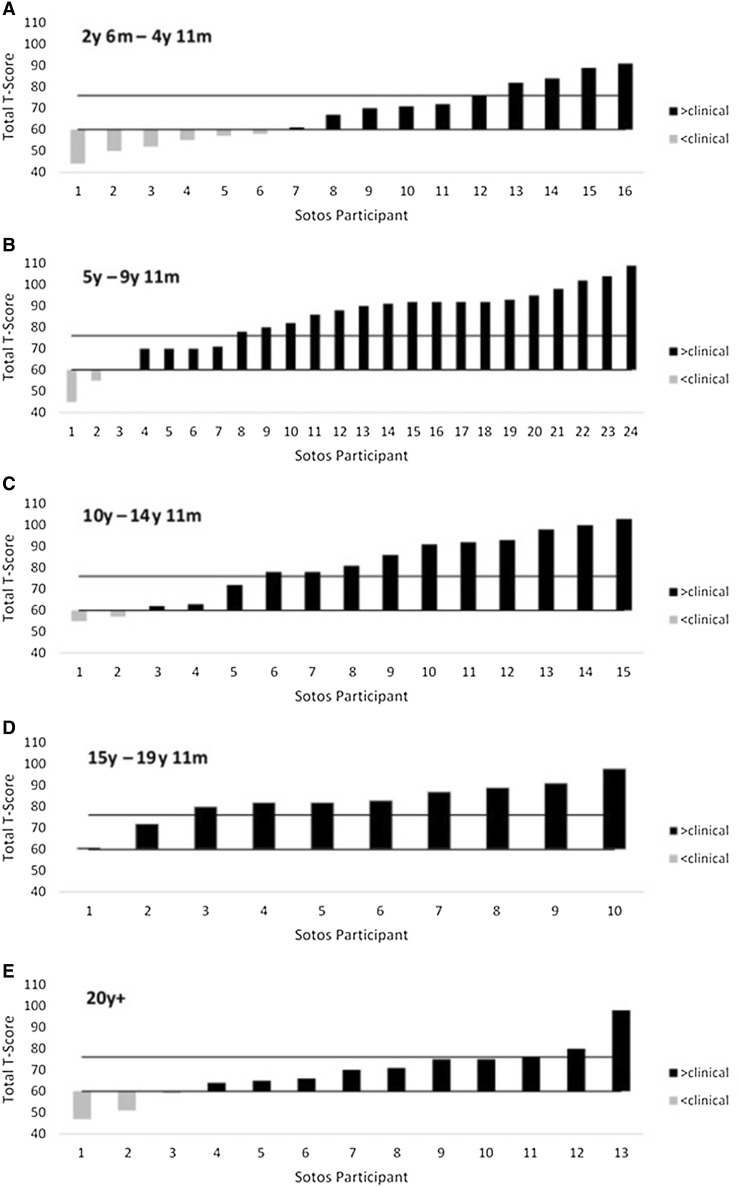
Waterfall plots of SRS-2 total T-scores by age category. In **a**–**e**, total T-scores are shown for Sotos individuals in distinct age categories: 2 years 6 months to 4 years 11 months, 5 years to 9 years 11 months, 10 years to 14 years 11 months, 15 years to 19 years 11 months and 20 years+, respectively. In each, the *lower line* depicts a T-score of 60. Scores *below this line* are non-clinical and scores *on or above this line* are in the mild and moderate symptom severity range. The *upper line* depicts a T-score of 76 and scores *on or above this line* are in the severe symptom severity range. In **a**, scores in the severe range were reported in 5 children (31.25 %). In **b**, scores in the severe range were reported in 17 children (70.83 %). In **c**, scores in the severe range were reported in 10 children (66.67 %). In **d**, scores in the severe range were reported in 8 individuals (80 %). In **e**, scores in the severe range were reported in 3 individuals (23.08 %)

In order to investigate the severity of symptoms across development, participants were categorised into 5 age groups: 2 years 6 months to 4 years 11 months (n = 16); 5 years to 9 years 11 months (n = 24); 10 years to 14 years 11 months (n = 15); 15 years to 19 years 11 months (n = 10) and 20 years and older (n = 13). A one-way ANOVA found a significant main effect of age category on total T-scores (*F*(4,77) = 4.88, *p* = .002). Specifically, this analysis identified that the model of best fit was quadratic (*F*(4,77) = 15.98, *p* < .001), indicating an inverted U-shaped pattern of total T-scores. Figure 2 shows that individuals with Sotos syndrome display ASD symptomatology which is less severe in early childhood (up to the age of 5 years) and adulthood, compared with childhood.

### DSM-5 Compatible Subscales

**Fig. 3:**
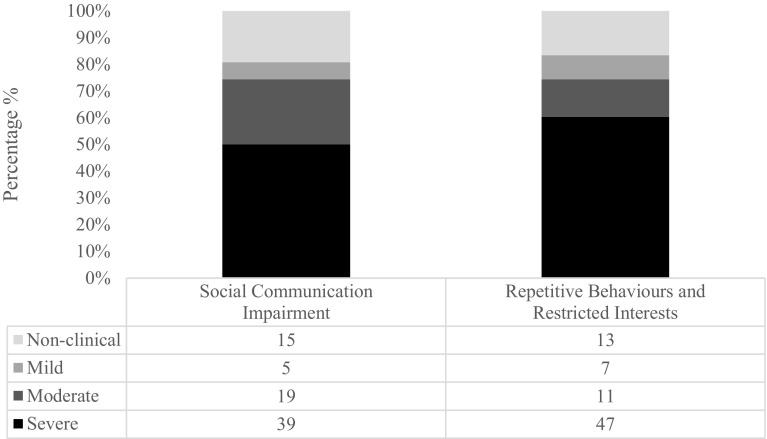
Distribution of severity of scores on each of the DSM-5 compatible subscales: social communication impairment and restricted interests and repetitive behaviours. The *numbers* represent total number of participants in each category

In order to investigate whether there were particular difficulties observed in either of the two DSM-5 domains, a paired-samples *t* test was used to compare scores on each of these subscales. The analysis identified a significant difference between T-scores on the social communication impairment (M = 75.57, SD = 15.43) and restricted interests and repetitive behaviours (M = 79.45, SD = 16.44) subscales, indicating that individuals with Sotos syndrome display greater difficulty with restricted interests and repetitive behaviours, compared with social communication impairment (*t*(77) = 4.37, *p* < .001). This was a large effect (*d* = 0.99). This is consistent with the profile of SRS-2 scores that is found in individuals with ASD and other clinical groups (Van Eeghen et al. 2013). Figure 3 shows the distribution of scores for the restricted interests and repetitive behaviours subscale and the distribution of scores for the social communication impairment subscale. The same categorisation of severity that was used for total T-scores was used for the subscales: non-clinical (T-score <60), mild (T-score of 60–65), moderate (T-score of 66–75) and severe (T-score ≥76).

### Subscale Analysis of Factors Identified from Frazier et al. (2014)

**Fig. 4:**
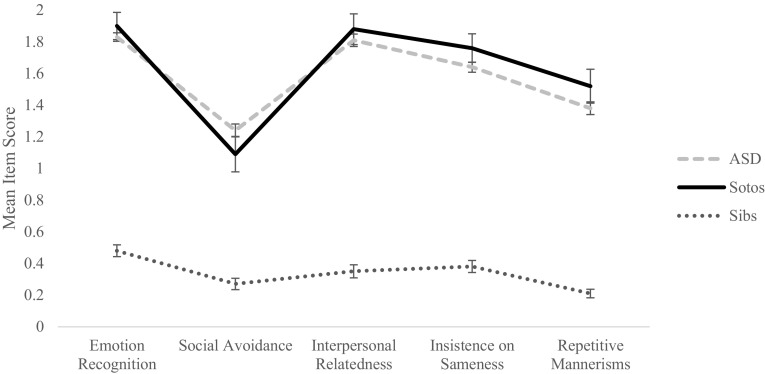
Mean item scores for the five subscales identified in the Frazier et al. (2014) factor analysis of the SRS-2. Data taken from (Frazier et al. 2014). *Error bars* show standard error

A recent factor analysis derived five empirical factors from the SRS-2: emotion recognition, social avoidance, interpersonal relatedness, insistence on sameness and repetitive mannerisms. The first three factors relate to social communication impairment and the remaining two factors relate to restricted interests and repetitive mannerisms (Frazier et al. 2014). The mean item scores and variance for each of the five factors from children with ASD (n = 271) and their unaffected siblings (n = 119), were taken from the Frazier et al. (2014) paper. In both the ASD and unaffected siblings groups, participants ranged in age from 4 to 18 years. These data were compared to our Sotos syndrome data. In order to provide a comparable sample, only our data from participants between 4 and 18 years of age were used for the Sotos syndrome group (n = 46). Average item scores of the Sotos syndrome children for each of the five factors were: emotion recognition (M = 1.90, SD = 0.58), social avoidance (M = 1.09, SD = 0.76), interpersonal relatedness (M = 1.88, SD = 0.66), insistence on sameness (M = 1.76, SD = 0.61) and repetitive mannerisms (M = 1.52, SD = 0.72). A 2 × 5 (Sotos/ASD × SRS subscale) mixed measures ANOVA found no main effect of diagnosis, (*F*(1,315) = 0.62, *p* = .43). There was also no significant group × subscale interaction, (*F*(4,1137) = 1.40, *p* = .23), demonstrating that children with Sotos syndrome appear to display a very similar symptom severity and profile of behaviour to that of children with ASD (see Fig. 4). By contrast, a 2 × 5 (Sotos/Sibs × SRS subscale) mixed measures ANOVA found a highly significant main effect of group, (*F*(1,163) = 474.88, *p* < .001) as scores for the children with Sotos syndrome were considerably higher than for the unaffected siblings. A significant group × subscale interaction, (*F*(4,606) = 18.37, *p* < .001), indicated that the behavioural profile was also different (see Fig. 4).

## Discussion

The primary aim of the present study was to investigate the prevalence and profile of autistic features in a large and representative sample of individuals with Sotos syndrome. Secondary aims of the study were to investigate the effects of age and gender on ASD symptom severity within the Sotos population. Within this study, 83.33 % of participants met clinical cut-off for ASD, as measured by the SRS-2. This finding suggests that the majority of individuals with Sotos syndrome display a current behavioural profile associated with the DSM-5 criteria for ASD (social communication impairment and restricted interests and repetitive behaviours). This indicates an important relationship between the behavioural phenotypes of Sotos syndrome and ASD.

Previous research has suggested relationships between other congenital syndromes and ASD. A recent systematic review and meta-analysis reported prevalence of ASD in a number of genetic syndromes with the highest estimate of 61 % identified in Rett’s syndrome (Richards et al. 2015). This particular review did not include Sotos syndrome, due to the fact that there is a lack of previous research investigating ASD within this population. However, it is clear from the findings in the present study that autistic symptomatology may be more prevalent in Sotos syndrome than many other genetic syndromes.

The reported prevalence of ASD symptomatology in Sotos syndrome in the present study is consistent with previous literature suggesting an association between Sotos syndrome and ASD (Lane et al. 2016; Sheth et al. 2015; Timonen-Soivio et al. 2016). Sheth et al. (2015) found that 26 of 38 participants (68.42 %) met clinical cut-off for ASD symptomatology, as assessed by the Lifetime form of the SCQ. However, as the present study found a significant effect of age and Sheth et al. (2015) used the Lifetime form to assess ASD symptomatology, which has a significant focus on the 4–5 years age range, this could account for the slightly higher prevalence identified in the present study. In addition, the profile of ASD symptomatology may be affected by age which could explain differences in the relative severity of impairment in social communication impairment and restricted interests and repetitive behaviours in the present study and the findings from Sheth et al. (2015).

In the present study, there was no effect of gender on symptom severity, indicating that there is no significant difference between the prevalence of behavioural characteristics associated with ASD in males and females with Sotos syndrome. This is an important finding as there is a significant gender difference in diagnosis of ASD, with males more likely to receive a diagnosis than females (Fombonne 2009). However, our findings indicate that severity of ASD symptomatology is comparable in both males and females with Sotos syndrome. It is important to note that within our sample, 16 participants had diagnoses of both Sotos syndrome and ASD, yet only two of these were females. This suggests that although males and females with Sotos syndrome appear to display a very similar behavioural phenotype, there is a clear disparity between diagnosis of ASD in males and females with Sotos syndrome.

The findings from the present study suggest that within the Sotos population, age affects severity of ASD symptomatology. Specifically, we found that ASD symptomatology was less severe in young children (2.5–5 years) and in adults (20+ years) when compared to children over the age of 5 years through to adolescence, in the current sample. This is an important finding as it suggests that severity of ASD symptomatology may decrease as an individual transitions into adulthood. Research investigating age-related effects of ASD symptomatology in individuals with idiopathic ASD indicates that the symptoms of ASD tend to abate, to some extent, in adolescence and young adulthood (Seltzer et al. 2004). Thus, findings from the present study are consistent with previous research investigating age-related effects in individuals with idiopathic ASD, indicating a similar trend within the Sotos population towards improvement in ASD symptomatology across the lifespan. However, as this study used a cross-sectional design, an important future direction will be to examine the effect of age using a longitudinal design, so that developmental trajectories can be effectively tracked.

It has been suggested that distinct profiles of ASD symptomatology may be associated with different genetic syndromes (Moss and Howlin 2009). The findings from the present study suggest that individuals with Sotos syndrome display trait profiles that are similar to those present in ASD. This is supported by the comparison of the Sotos syndrome and ASD data on the five empirically derived subscales identified by the recent factor analysis of the SRS-2 (Frazier et al. 2014). Children with Sotos syndrome appear to display behavioural characteristics of a similar profile and severity to that identified in ASD and were distinct from scores identified in the unaffected siblings of the ASD children. However, as this study measured autistic features using a questionnaire, it will be important for future research to explore the profile of ASD symptomatology in Sotos syndrome in more detail, using clinical evaluations, such as the Autism Diagnostic Observation Schedule (Lord et al. 2000) and a matched control group of individuals with ASD. A limitation of the present study is that information such as the developmental level of the participants was not collected. Thus, it will be useful for future research to investigate factors which may affect ASD symptomatology within this population, such as developmental level, cognitive ability and verbal ability. This will enhance understanding of the behavioural phenotype of Sotos syndrome.

To date, research investigating ASD in Sotos syndrome has focused on the prevalence of behavioural characteristics. It will also be beneficial for future research to examine areas such as theory of mind, executive functioning and cognitive abilities, as these have not yet been investigated within the Sotos population, to establish whether the cognitive profile is similar or distinct from ASD. This will enhance understanding of the broader phenotype of Sotos syndrome and enable development of interventions and educational programmes that are targeted specifically for the Sotos population.

## Conclusion

In summary, this is the largest study to date to investigate symptomatology associated with ASD in individuals with Sotos syndrome. The findings from the present study demonstrate a high prevalence of autistic symptomatology within the Sotos population and suggest that the majority of individuals with Sotos syndrome display clinically significant behavioural symptomatology associated with ASD. Symptom severity does not appear to be affected by gender but does seem to differ in relation to age, with more prominent behavioural characteristics in childhood (5–19 years), compared with early childhood (2.5–5 years) and adulthood (20 years and older). As the majority of cases of Sotos syndrome are caused by abnormality of the NSD1 gene, our findings provide further evidence to suggest a possible genetic mechanism associated with ASD. An important clinical implication of our findings is that clinicians should screen for ASD in individuals with Sotos syndrome as there may be a number of unidentified cases of co-morbidity.
